# Discovery of Gramine Derivatives That Inhibit the Early Stage of EV71 Replication *in Vitro*

**DOI:** 10.3390/molecules19078949

**Published:** 2014-06-27

**Authors:** Yanhong Wei, Liqiao Shi, Kaimei Wang, Manli Liu, Qingyu Yang, Ziwen Yang, Shaoyong Ke

**Affiliations:** 1 College of Life Sciences, Wuhan University, Wuhan 430072, China; E-Mails: weiyanhong925@163.com (Y.W.); yangqingyu8671@163.com (Q.Y.); 2 National Biopesticide Engineering Research Center, Hubei Academy of Agricultural Sciences, Wuhan 430064, China; E-Mails: shiliqiao_75@163.com (L.S.); wang_kaimei@163.com (K.W.); newmoney@163.com (M.L.)

**Keywords:** antiviral activity, enterovirus 71, gramine derivatives

## Abstract

Enterovirus 71 (EV71) is a notable causative agent of hand, foot, and mouth disease in children, which is associated with an increased incidence of severe neurological disease and death, yet there is no specific treatment or vaccine for EV71 infections. In this study, the antiviral activity of gramine and 21 gramine derivatives against EV71 was investigated in cell-based assays. Eighteen derivatives displayed some degree of inhibitory effects against EV71, in that they could effectively inhibit virus-induced cytopathic effects (CPEs), but the anti-EV71 activity of the lead compound gramine was not observed. Studies on the preliminary modes of action showed that these compounds functioned by targeting the early stage of the EV71 lifecycle after viral entry, rather than inactivating the virus directly, inhibiting virus adsorption or affecting viral release from the cells. Among these derivatives, one (compound **4s**) containing pyridine and benzothiazole units showed the most potency against EV71. Further studies demonstrated that derivative **4s** could profoundly inhibit viral RNA replication, protein synthesis, and virus-induced apoptosis in RD cells. These results indicate that derivative **4s** might be a feasible therapeutic agent against EV71 infection and that these gramine derivatives may provide promising lead scaffolds for the further design and synthesis of potential antiviral agents.

## 1. Introduction

Enterovirus 71 (EV71) is a single positive-stranded RNA virus that belongs to the *Enterovirus* genus of the Picornaviridae family. It was first isolated and characterized in cases of neurological disease in the United States in 1969 [1]; subsequent outbreaks of EV71 infections have been reported around the world in the past decades, especially in the Asia-Pacific region in countries like Malaysia [2], Australia [3], Germany [4], Japan [5], the United Kingdom [6], Taiwan [7] and mainland China [8,9]. EV71 infections predominantly cause hand, foot, and mouth disease (HFMD) or herpangina and are typically found in infants and children, where they are associated with nervous system diseases, ranging from aseptic meningitis to fatal encephalitis [10,11]. According to reports from the Chinese Center for Disease Control and Prevention, HFMD was listed as the most common category-C infectious disease from 2009 to 2011, based on incidence and death rate, with more than 500 deaths in over 1,600,000 cases of EV71 infection reported in China in 2011 alone [12].

There is currently no vaccine or specific medication for EV71 infections [12], highlighting the urgency and significance of developing suitable anti-EV71 agents. At present, the prevention of EV71 epidemics mainly depends upon public surveillance. Ribavirin, type I interferon, and pleconaril have been used to treat EV71 infections [13,14,15]; some compounds also showed activity against EV71 in both cell lines and animal models, but a clinical application is not yet available, so more effort should be made to develop drugs to conquer EV71 infections.

Many compounds from various pharmacological medicinal plants have been extensively researched, not only for their potential inhibitory properties against virus invasion, but also for their low toxicity in cells. Gramine, a natural indole alkaloid, has been isolated from various raw plants and coal tar, and exhibits broad pharmaceutical activities, such as relaxation of bronchial smooth muscle, vasorelaxation, blood pressure elevation, relief of bronchitis nephritis, and bronchial asthma [16]. Up to now, gramine has been widely used as a pharmaceutical lead scaffold for constructing various biologically active indole-containing compounds [17,18,19]. Many indole-type analogs have already been synthesized by different routes with various improvements in biological activity [20,21,22]. We have reported previously that a series of novel gramine derivatives showed potential anticancer activity *in vitro* [23], which inspired us to investigate their antiviral activity for use as an effective treatment for EV71 infections. Herein, we report the discovery of gramine derivatives that act as inhibitors of EV71 infection *in vitro* and the preliminary modes of action of these derivatives against EV71.

## 2. Results

### 2.1. Antiviral Activity of Gramine and Its Derivatives

**Figure 1:**
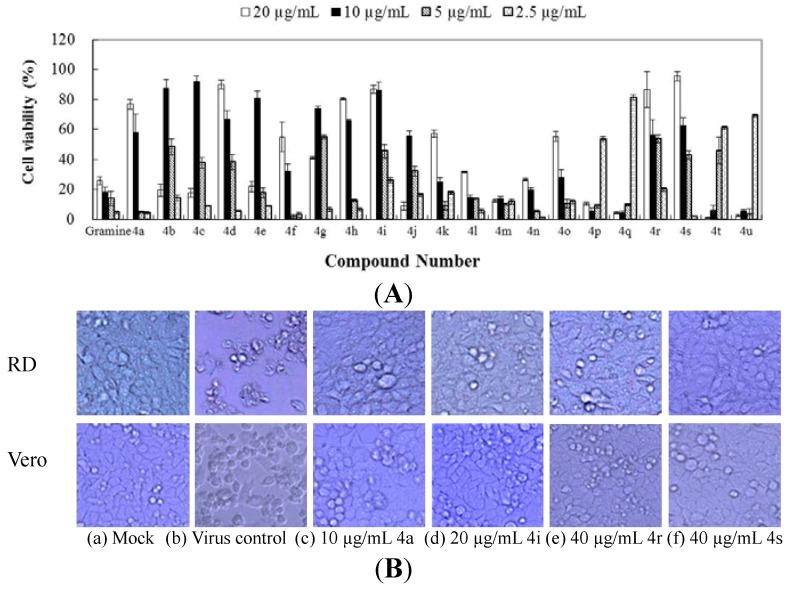
Gramine and its synthetic derivatives inhibited virus-induced CPE. (**A**) Vero cells were infected with 100 TCID_50_ of EV71 mixed with serial dilutions of tested compounds for 1.5 h at 37 °C; the inoculum was aspirated and cells were incubated with DMEM/tested compounds at 37 °C, 5% CO_2_ for 48 h pi. The viability of the cells was determined with an MTT assay; **(B)** Morphology image of Vero and RD cells treated with compounds 4a, 4i, 4r, and 4s for 48 h pi. (magnification, 20×).

The antiviral activities of gramine and its derivatives against EV71 based on inhibition of virus-induced cytopathogenicity effects (CPEs) in African green monkey kidney cells (Vero) and rhabdomyosarcoma cells (RD) were examined. The cytotoxic effects were also evaluated. The inhibitory activities expressed as half maximal effective concentration (EC_50_) values and selectivity indexes (SI) for the target compounds are presented in Table 1, and the dose-dependent antiviral effects are shown in Figure 1A.

**Table 1:** Cytotoxicity and Antiviral Activity of Gramine and its Synthetic Derivatives against Enterovirus 71 (EV71).

Compound	Structural Formula	EC_50_ (µg/mL) ^a^		CC_50_ (µg/mL) ^b^		SI ^c^	
		Vero	RD	Vero	RD	Vero	RD
**Gramine**	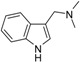	— ^d^	—	101.4 ± 5.6 ^e^	85.0 ± 16.3	—	—
**4a**	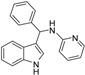	12.7 ± 3.2	5.5 ± 2.4	32.6 ± 3.5	17.2 ± 1.6	2.6	3.1
**4b**	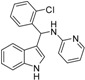	5.1 ± 2.7	7.4 ± 1.1	18.6 ± 1.4	22.8 ± 1.7	3.6	3.1
**4c**	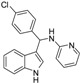	5.6 ± 1.3	6.7 ± 0.8	22.4 ± 3.6	24.6 ± 3.5	4.0	3.7
**4d**	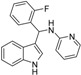	9.2 ± 2.1	5.8 ± 1.4	37.8 ± 5.8	28.0 ± 5.2	4.1	4.8
**4e**	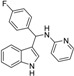	7.3 ± 2.6	6.2 ± 2.8	40.7 ± 2.5	29.5 ± 3.3	5.6	4.8
**4f**	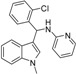 7	14.4 ± 3.2	—	86.4 ± 7.4	44.0 ± 5.1	6.0	—
**4g**	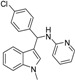	4.9 ± 0.7	4.9 ± 1.2	38.6 ± 5.7	32.0 ± 1.8	7.9	6.5
**4h**	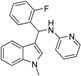	10.5 ± 2.1	11.2 ± 4.6	89.3 ± 12.6	52.2 ± 8.3	8.5	4.7
**4i**	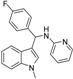	5.5 ± 1.6	6.1 ± 3.2	71.1 ± 3.8	65.2 ± 7.6	12.9	10.7
**4j**	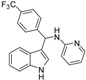	8.7 ± 2.9	8.3 ± 3.5	15.8 ± 3.3	17.8 ± 2.6	1.8	2.1
**4k**	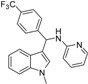	13.2 ± 1.3	9.8 ± 3.4	18.5 ± 4.8	27.6 ± 8.3	1.4	2.8
**4l**	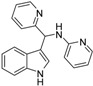	—	—	96.2 ± 15.5	65.2 ± 9.1	—	—
**4m**	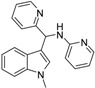	—	—	>200	>200	—	—
**4n**	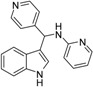	—	—	120.7 ± 20.5	>200	—	—
**4o**	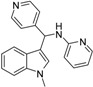	16.9 ± 1.5	9.5 ± 1.7	72.8 ± 18.8	39.2 ± 11.6	4.3	4.1
**4p**	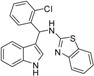	2.1 ± 0.7	3.2 ± 0.4	6.6 ± 1.4	11.7 ± 4.6	3.1	3.7
**4q**	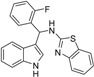	1.8 ± 1.1	2.7 ± 1.2	10.8 ± 2.5	14.5 ± 3.6	6.0	5.4
**4r**	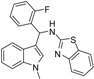	9.9 ± 1.4	7.3 ± 1.3	132.5 ± 8.3	149.9 ± 16.2	13.4	20.5
**4s**	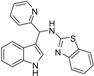	7.6 ± 2.5	9.1 ± 3.1	108.7 ± 16.8	136.7 ± 9.3	14.3	15.0
**4t**	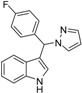	1.9 ± 0.6	2.6 ± 0.8	10.5 ± 2.4	12.6 ± 3.5	5.5	4.8
**4u**	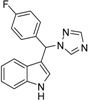	2.3 ± 0.3	3.1 ± 1.3	4.9 ± 0.6	6.8 ± 2.5	2.1	2.2
**ribavirin ^f^**	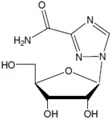	44.6 ± 13.5	32.1	>500	435.8	>11	13.6

^a^ EC_50_, compound concentration required to achieve 50% protection from virus-induced cytopathogenicity; ^b^ CC_50_, compound concentration required to reduce cell viability by 50%; ^c^ SI (Selectivity index), ratio CC_50_/EC_50_; ^d^ less than 50% inhibition; ^e^ Values represent the mean ± standard deviation of three independent experiments; ^f^ ribavirin, used as a positive control.

It was observed that 18 synthetic gramine derivatives displayed some degree of inhibitory effect against EV71-induced CPEs compared with a virus control, with EC_50_ values in the 1.8–16.9 µg/mL range and corresponding SI values of 1.4–20.5, while the anti-EV71 activity of the lead compound gramine was not observed (Table 1, Figure 1A). It seems that the incorporation of heterocyclic units would be favorable for the derivatives’ antiviral activities against EV71, and changes in substituents on the heterocyclic unit and other combinations of functional groups also affect the antiviral effects. Compounds **4b** to **4e** were endowed with significant activities against EV71, similar to compound **4a**, but were toxic. The introduction of a methyl group into the parent gramine unit (compounds **4f** to **4h**) modified the cytotoxicity of these compounds, while maintaining different degrees of antiviral activity, with better SI. Generally, compounds bearing a methyl moiety in the parent gramine unite have reduced cytotoxic effects (cytotoxicity: **4b** < **4f**, **4c** < **4g**, **4d** < **4h**, **4e** < **4i**, **4j** < **4k**, **4l** < **4m**, **4q** < **4r**), which leads to a potential increase in SI. The presence of -CF_3_ groups on a benzene unit (**4j**, **4k**) decreased anti-EV71 activity and enhanced toxicity, with the smallest SI compared with other active compounds. Compounds carrying pyridin-4-yl and pyridin-2-yl substituents (**4l** to **4o**) were devoid of activity and toxicity, with the exception of **4o**, which had a slight inhibitory effect against EV71. Thus, the anti-EV71 activities of compounds **4p** to **4s** were markedly increased by the introduction of a benzothiazole moiety; among these, **4p** and **4q** exhibited considerable cytotoxic effects. However, for compounds **4r** and **4s**, the anti-EV71 activities were also coupled with low cytotoxicity, resulting in high therapeutic indexes. In particular, compound **4s**, containing pyridine and benzothiazole units, showed the strongest potency with an EC_50_ value of 7.6 µg/mL in Vero cells and 9.1 µg/mL in RD cells, and corresponding SI of 14.3 in Vero cells and 15.0 in RD cells relative to other synthetic derivatives. The compounds **4t** bearing a pyrazole unit and **4u** containing a 1,2,4-triazole unit exhibited strong cytotoxicity and good antiviral effects. The inhibition effects of virus-induced CPEs of representative compounds **4a**, **4i**, **4r**, and **4s** are shown in Figure 1B. The EV71-infected cells had a rounded appearance and were detached from the dish in the absence of test compounds (Figure 1B-b), while a nearly complete inhibition of CPE was observed when RD cells were treated with 10 µg/mL **4a**, 20 µg/mL **4i**, or 40 µg/mL **4r** or **4s** (Figure 1B c–f), which was used for the subsequent experiments in this study.

### 2.2. Preliminary Studies on Mechanisms of Action: Gramine Derivatives Mainly Target the Post-Infection Stage

Based on structure specificity, antiviral activity, and selectivity therapeutic index, compounds **4a**, **4i, 4r** and **4s** were chosen for further investigation on modes of action against EV71 infection. To determine whether these gramine derivatives inactivated virions directly, 10^4^ TCID_50_ (median tissue culture infective dose) of EV71 suspension was incubated with 20 µg/mL **4a**, 40 µg/mL **4i**, or 80 µg/mL **4r** or **4s** for 24 h at 4 °C; subsequently, the virus titers in the mixture were measured by inoculating 100-fold dilution of the mixtures beyond the effective concentrations of the compounds into the host cells. The TCID_50_ values were calculated by the Reed-Muench method [24] based on the CPE observed on day 2 post inoculation (pi). No significant difference in virus titers was found between the mixture with and without the tested compounds for EV71 (data not shown). Thus, these compounds appear not to be virucidal for the viruses tested.

**Figure 2:**
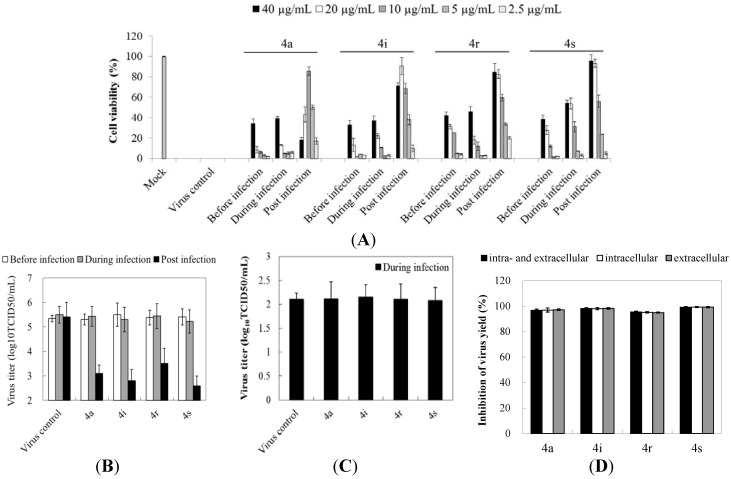
Analysis of modes of action against EV71 of gramine derivatives. Analysis of effective stage. The RD cells were treated with tested compounds before, simultaneously or after EV71 (100 TCID_50_) inoculation: antiviral effects were detected by measuring (**A**) cell viability and (**B**) progeny virus yields (10 µg/mL **4a**, 20 µg/mL **4i**, 40 µg/mL **4r** and **4s**) 48 h post infection; (**C**) Analysis of the effects on EV71 adsorption. Mock- or 10 µg/mL **4a**, 20 µg/mL **4i**, 40 µg/mL **4r** and **4s**-treated EV71 (10^4^ TCID_50_) was spinoculated onto RD cells and adsorbed for 2 h; intracellular virus was subjected to titration using the TCID_50_ method; (**D**) Effects on EV71 release from RD cells. RD cells infected with 100 TCID_50_ of EV71 were incubated with 10 µg/mL **4a**, 20 µg/mL **4i**, 40 µg/mL **4r** and **4s** for 12 h, of both cells and supernatants (intra- and extracellular), or of cells or supernatants harvested separately for virus titration. The figure shows the inhibition rate of progeny virus yield compared with the virus control. Values represent the mean ± standard deviation of three independent experiments.

To identify the step in the viral life cycle that is affected by these compounds, the assays were performed using three different treatment protocols. (i) To analyze for a preventive effect (before infection), the compounds were added to cells for 2 h at 37 °C followed by washing with maintenance medium (MM) before virus infection; (ii) to analyze for inhibition of adsorption (during infection), the mixtures of the compounds and virus were added to cells for 2 h at 37 °C followed by washing with MM; (iii) to analyze for a therapeutic effect (post infection), the cells were first infected for 1 h at 37 °C followed by washing with MM, the compounds **4a**, **4i**, **4r**, and **4s** were added and incubated with the cells for the duration of the experiment. For all treatments, cell viability and progeny virus yields were measured after 48 h of infection. As shown in Figure 2A,B, the compound-pretreated cells were not fully resistant to subsequent infections of EV71 compared with the control group (Figure 2A). The titers of virus from the infected cells treated with 10 µg/mL **4a**, 20 µg/mL **4i**, or 40 µg/mL **4r **or **4s** were nearly the same as those from untreated cells at 48 h (Figure 2B), indicating that these compounds have no obvious effects on cellular function to prevent virus infection.

Similar results were obtained when the compounds were added to RD cells concurrently with EV71; there was no apparent inhibitory effect on virus-induced CPE and progeny virus yield at 48 h (Figure 2A,B), indicating that EV71 adsorption was not inhibited by these gramine derivatives. These results were also confirmed by intracellular virus titration using the TCID_50_ method. Mock- or 10 µg/mL **4a**, 20 µg/mL **4i**, or 40 µg/mL **4r** or **4s**-treated EV71 (10^4^ TCID_50_) were spinoculated onto cells and adsorbed for 2 h. The infected cells were harvested following freeze–thaw cycles and then subjected to virus titration. As can be seen in Figure 2C, no significant decrease in virus titer during virus attachment in the presence of these derivatives was detected, confirming that virus adsorption is not quantitatively affected by these compounds.

All of the tested compounds exhibited the most powerful therapeutic effects in a dose-dependent manner. The viability rates of the infected cells treated with 10 µg/mL **4a**, 20 µg/mL **4i**, or 40 µg/mL **4r** or **4s** were 85.8%, 90.5%, 84.6%, and 95.7% (Figure 2A). For compound **4a**, no inhibition of CPEs at the 40 µg/mL and 20 µg/mL levels was exhibited, due to its cytotoxic effects on cells, while compounds **4r** and **4s** almost completely inhibited virus-induced CPEs, and no morphological cytotoxicity was observed for these compounds up to a concentration of 40 µg/mL (Figure 2A), reflecting a significant increase in SI. The titers of virus from compound-treated cells were also much lower than those from untreated cells (Figure 2B). These results indicate that these gramine derivatives mainly block the post-attachment stage of the EV71 infection.

A possible effect of the compounds on viral release from cells was tested. The RD cells were treated with or without 10 µg/mL **4a**, 20 µg/mL **4i**, or 40 µg/mL **4r** or **4s** after EV71 infection, and the supernatants and cells were harvested together or separately for analysis of progeny virus yields at 12 h. Figure 2D shows that the inhibition rates of virus yields for these compounds were completely consistent with the supernatants or total solutions (cell lysis solutions and the supernatants), implying that these gramine derivatives have no effect on the release of EV71.

These results suggest that gramine derivatives **4a**, **4i**, **4r** and **4s** show neither preventive effects against EV71 nor inhibiting adsorption of EV71, have no effects on release of EV71, but mainly block the post-attachment stage of the viral infection.

### 2.3. Gramine Derivatives Affect Viral Early Steps of Replication in Cells

**Figure 3:**
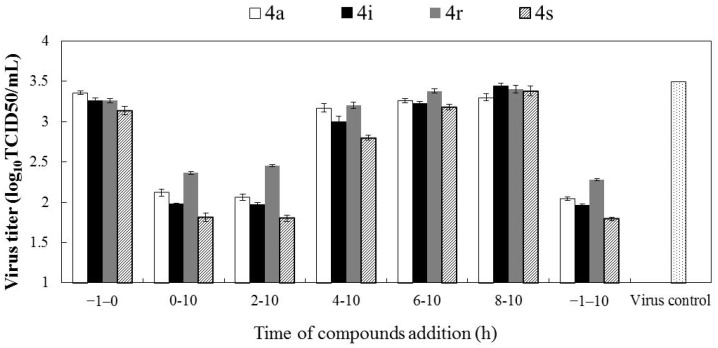
Time-of-addition assay. 10 µg/mL **4a**, 20 µg/mL **4i**, or 40 µg/mL **4r** or **4s** were added to RD cells at different time periods after EV71 infection. At 12 h pi, the progeny virus yield was determined (−1–0 h: viral infection period; 0–10 h: period for virus proliferation in the cells). Values are represented as the mean ± standard deviation.

To further understand the mechanisms of these gramine derivatives against EV71 propagation in cells, a time-of-addition experiment was performed. For this experiment, 10 µg/mL **4a**, 20 µg/mL **4i**, or 40 µg/mL **4r** or **4s** were added to infected cultures at different time periods (with 2 h intervals) and the reductions in virus yield relative to untreated cultures were determined at 12 h pi. As shown in Figure 3, when the compounds were present for the whole course of the replication cycle (-1-10 h pi), the titers of the progeny virus were strongly reduced, which was similar to that for the addition of drugs during the 0–10 h and 2–10 h stages after viral infection (pi). With drugs treatments during other periods after EV71 infection, a gradual increase of viral yields was observed, reflecting the loss of the antiviral effects of the compounds. Results from this experiment indicate that these gramine derivatives may exert antiviral effects via the same action mechanism and mainly interfere with the early events of virus replication.

### 2.4. Derivative **4s** Inhibits Strongly Viral Replication in RD Cells

**Figure 4:**
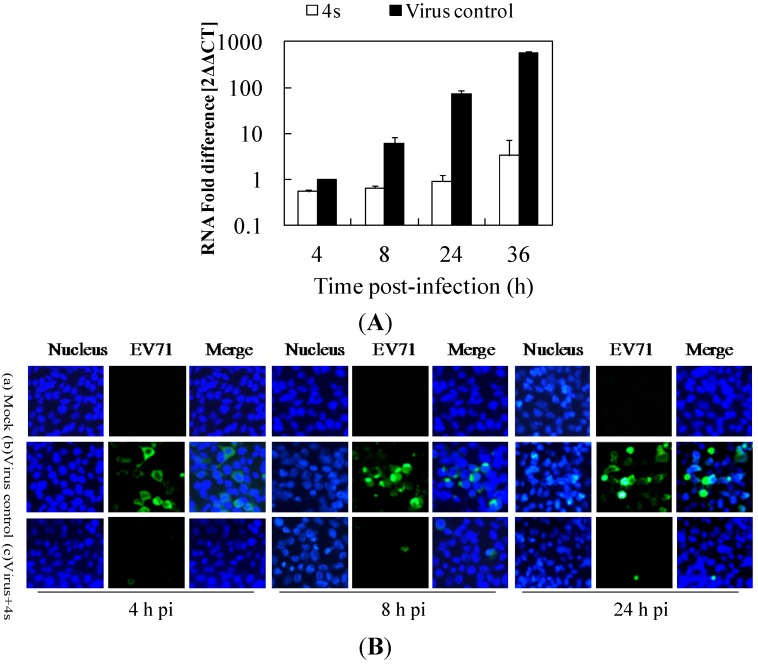
The effect of **4s** on EV71 replication in RD cells. RD cells infected with 100 TCID_50_ of EV71 were incubated in the absence (virus control) or presence of 40 µg/mL **4s** and harvested at the indicated times pi. (**A**) Total RNA was extracted from cells and culture supernatants and EV71 RNA levels were measured. Cellular actin amplification was used for normalization. The ΔΔCt data were calculated from three independent experiments; (**B**) EV71-protein was determined by indirect immunofluorescence using a mouse anti-enterovirus 71 monoclonal antibody and an Alexa-Fluor-488-conjugated AffiniPure goat anti-mouse IgG (H + L). The nucleus was stained with DAPI; the green foci indicate the presence of EV71 protein.

Derivative **4s** showed the most potency against EV71 in a first set of experiments. To further analyze the effect of **4s** on EV71 replication in RD cells, the efficacy of **4s** in inhibiting viral RNA synthesis was analyzed by quantitative reverse transcription polymerase chain reaction (RT-PCR). The infected cells treated with or without 40 µg/mL **4s** were harvested at 4, 8, 24 and 36 h pi to examine the relative amount of viral RNA. The fold difference of RNA for all the samples was calculated relative to the RNA level in the EV71-infected cells (virus control) at 4 h pi. Figure 4A shows that the level of viral RNA in the virus control group became detectable in the first 4 h and was followed by a significant increase at other time points. The viral copy numbers in the **4s**-treated RD cells were significantly lower than those in the virus control cells, with fold reductions of 1.8, 9.0, and 79.8 at 4 h, 8 h, and 24 h pi. The inhibitory effect was most prominent at 36 h, with a 174.7-fold reduction. These data clearly confirm that derivative **4s** inhibits strongly viral replication in RD cells.

The influence of **4s** on the replication of EV71 was also determined at the translational level. The RD cells grown in 24 coverslips were infected with EV71 and treated with 40 µg/mL **4s** post-infection, intracellular viral protein was detected by indirect immunofluorescence analysis after incubation for 4, 8 or 24 h. No immunofluorescent foci of viral protein were observed in the mock-infected control (Figure 4B-a), which suggested that the antibody was specific for EV71. The green immunofluorescent foci were significantly more abundant in the virus control group (Figure 4B-b) than in the **4s**-treated cells (Figure 4B-c), which indicated that the viral protein synthesis was suppressed by **4s** as a result of a cumulative inhibitory action on viral RNA synthesis.

### 2.5. Compound **4s** Inhibits EV71-Induced Apoptosis

**Figure 5:**
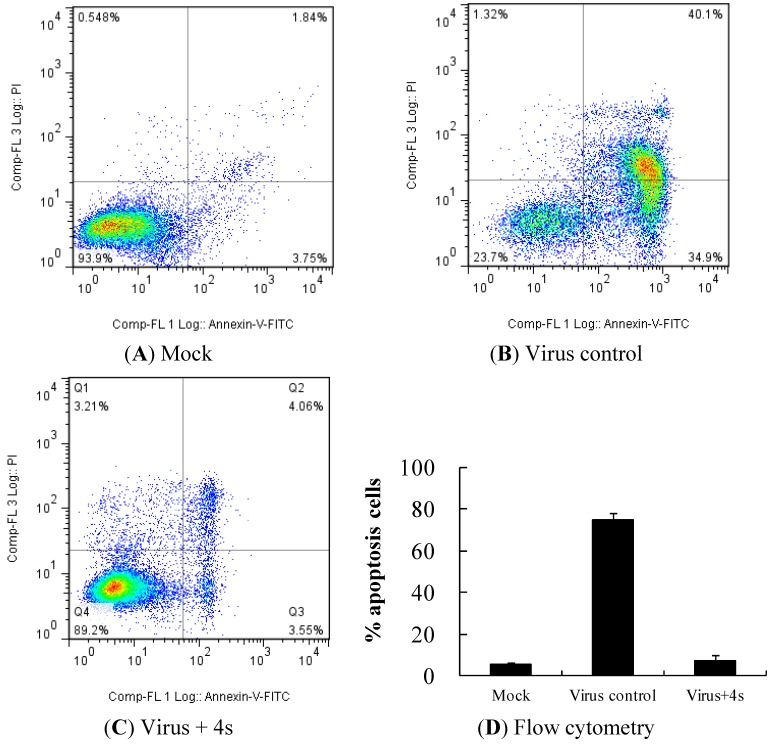
Inhibition effect of 4s on EV71-induced apoptosis. RD cells were (**A**) left untreated or (**B**) infected with 100 TCID_50_ of EV71. After viral adsorption, RD cells were incubated in the (**B**) absence or (**C**) presence of 40 µg/mL 4s for 36–48 h; (**D**) The cells were stained with Annexin-V- fluorescein and propidium iodide and measured using flow cytometry.

Previous studies showed that EV71 infection induces cell death by apoptosis [25]. EV71 adsorption, internalization, entry, uncoating, and viral RNA replication are not required to trigger infected cell apoptosis, but it is required for viral protein synthesis [26]. Flow cytometry was performed to investigate the effect of **4s** on virus-induced cell apoptosis. In the assays, EV71-infected cells were stained with Annexin-V-fluorescein and propidium iodide; fluorescence drifting indicates cells undergoing apoptosis. The RD cells infected with EV71 (virus control) showed a significant fluorescence drift to the right (representative of early apoptosis) and to the upper-right quadrant (representative of late apoptosis or death) (Figure 5B) in comparison with control cells (Figure 5A), while fluorescence drifting could hardly be observed with the addition of **4s** (Figure 5C). Compound **4s** could effectively inhibit EV71-induced apoptosis in RD cells, which indirectly indicates its function for inhibiting viral protein synthesis.

## 3. Discussion

The parent compound gramine did not exhibit anti-EV71 activity; however, the gramine derivatives have been demonstrated to be effective inhibitors of EV71 replication *in vitro*. They also inhibited coxsackievirus B3, adenovirus type 7, and herpes simplex virus 1 (data not shown), demonstrating a broad antiviral activity. Thus, our findings described here will support further study and the design of new more effective derivatives. The structural differences of these gramine derivatives from other antiviral agents should help in determining the feasibility of developing novel compounds for treatment of EV71; the present compounds contain novel unexplored structural features, whose relevance in the antiviral field deserves further investigation.

We recently reported the *in vitro* anticancer activity of these gramine derivatives [23], thus demonstrating that this set of substances possesses a certain degree of toxicity. Indeed, all tested compounds were certainly more toxic than the reference drug ribavirin (Table 1); however, they could inhibit the replication of EV71 at lower concentrations. For derivatives **4r** and **4s**, the SI values (20.5, 15.0) were equivalent to or better than the control compound ribavirin (13.6) in RD cells (Table 1). This provides evidence that the compounds exhibit cytotoxic effects on the host cells after playing an antiviral role rather than destroying cells directly to inhibit virus proliferation in them. Moreover, the novelty of this molecular structure for antiviral activity is noteworthy and, certainly, deserves further detailed investigations to find new compounds with a more favorable ratio between activity and toxicity.

Although not all the details of EV71 replication are understood, all enteroviruses do share the similar viral genome structure and replication strategy [27]. The EV71 replication cycle can be divided into the following steps: viral attachment, uncoating, entry, polyprotein translation and cleavage, viral RNA replication, viral assembly, and release. These critical steps are currently considered the targets for antiviral development [28]. The kinetics of picornavirus replication are rapid; they could complete their lifecycle in 5–10 h (approximately 8 h). Genomic RNA is translated directly by polysomes; the viral protein synthesis is predicted to reach high levels at 3–4 h pi; assembly begins in the cytoplasm during 4–6 h pi; and the release of virus particles is conducted during 6–10 h [29]. For these gramine derivatives, the time-addition assay showed that the inhibition rates of virus yield declined sharply with drug treatment at more than 4 h pi, which was consistent with EV71 protein synthesis at a high level during 3–4 h pi. Therefore, we propose that these gramine derivatives appear to act mainly on the early stage of EV71 proliferation, including viral RNA and protein synthesis after infection. More detailed analyses of the mechanisms of action of these compounds are currently being conducted.

EV71-induced apoptosis has been considered an important mechanism in disease pathogenesis [30]. Apoptosis leads to the spread of viral progeny, which may cause viremia and severe central nervous system complications. In this study, the compound **4s** was found to inhibit EV71-induced apoptosis, which may have a significant impact in protecting the host from severe consequences associated with infection with EV71.

Compound **4s** showed potent post-exposure activity on EV71-infected cells. This activity was initially tested by effective stage analysis (determining the viability of the cells) and time-of-addition experiments (measuring progeny viral yield), and was further confirmed by inhibition of viral RNA and protein synthesis and virus-induced cell apoptosis assay. This provides strong evidence that compound 4s is suitable for use as a therapeutic agent against EV71 infection.

In summary, we have found that a series of gramine derivatives exhibited selective inhibitory effects against EV71 infections and we have preliminarily elucidated the inhibition mechanisms of these compounds. Additional work will be carried out to investigate the potential for developing derivative 4s into an effective antiviral treatment for EV71 infections, for example, by studying the effect on virus model mice. Moreover, the modification of the gramine structure provides new insights into the development of drugs effective against virus infection.

## 4. Experimental

### 4.1. Cells and Viruses

Human RD cells and Vero cells were maintained in Dulbecco’s modified Eagle’s medium (Gibco-Invitrogen, Carlsbad, CA, USA) supplemented with 10% fetal bovine serum (FBS; Gibco), 100 U/mL of penicillin and streptomycin, and 2 mM L-glutamine. Enterovirus 71 (EV71) was kindly provided by Zhanqiu Yang (Institute of Medical Virology, School of Medicine, Wuhan University, China) and propagated in RD cell lines. The virus titer was determined by the standard method of median tissue culture infective dose (TCID_50_) on RD cells [24]. 

### 4.2. Preparation of the Tested Compounds

The gramine analogs **4a** to **4u** were synthesized in our laboratory according to previously reported methods [23]. The formulas of the test compounds are presented in Table 1. Drug stock solutions were prepared in dimethyl sulfoxide (DMSO) with a final concentration of 0.1%, which did not affect the biological assay results, and diluted with MM consisting of DMEM with 2% FBS.

### 4.3. Cytotoxicity and Antiviral Activity

The activity of compounds against EV71 was determined based on the inhibition of virus-induced cytopathogenicity effects (CPEs) in acutely infected cells. Vero and RD cells grown to 90% confluency in 96-well dishes were washed with phosphate buffered saline (PBS) and infected with 100 TCID_50_ of EV71 mixed with serial dilutions of tested compounds for 1.5 h at 37 °C. The inoculum was aspirated and cells were incubated with DMEM and test compounds at 37 °C, 5% CO_2_ for 48 h. The CPEs were observed microscopically and the viability of the cells was determined with a 3-(4,5-dimethylthiazol-2-yl)-2,5-diphenyltetrazolium bromide (MTT) assay. The concentrations required for the test compounds to achieve 50% protection from virus-induced cytopathogenicity (EC_50_) were determined. Adverse effects of these compounds on the host cells were also assessed by means of the MTT-method; by exposing uninfected cells to various concentrations of test compounds for 48 h at 37 °C, the viability of the cells was detected. The 50% cell cytotoxic concentrations (CC_50_) of compounds were calculated with the SPSS software (SPSS, Chicago, IL, USA). Selectivity indexes were calculated as the ratio of CC_50_: EC_50_.

### 4.4. Determination of Cell Viability

Cell viability was assessed by an MTT assay, which functions based on the reduction of a MTT into formazan dye by active mitochondria. The cells were treated with 100 µL of MTT (1 mg/mL, Sigma, St. Louis, MO, USA) and incubated at 37 °C for 4 h. The reaction was blocked by DMSO and measured in a microplate reader (Bio-Tek Instruments, Winooski, VT, USA) at 492 nm. The untreated control was arbitrarily set as 100%.

### 4.5. Virus Yield Reduction Assay

The virus suspension, serially diluted 10-fold with DMEM containing 2% FBS, was inoculated to cells in a 96-well plate. After 1 h incubation at 37 °C in 5% CO_2_, unbinding virus was washed out and MM was added to the cells. After 2 days, the infected cells were monitored for cytopathic effects (CPEs). The virus titer was calculated by the Reed–Muench method [24].

### 4.6. Time-of-Addition Assay

Time-of-addition experiments were carried out, to estimate the point where the compound interfered with the replication cycle of EV71. The RD cells were infected with 100 TCID_50_ of EV71 and then 10 µg/mL 4a, 20 µg/mL 4i, or 40 µg/mL 4r or 4s was added at different time phases (−1–0 h, 0–10 h, 2–10 h, 4–10 h, 6–10 h, 8–10 h and −1–10 h pi, where −1–0 h is the viral infection period and 0–10 h is the period for virus proliferation in the cells). The cells and supernatants were harvested at 10 h post infection (pi) and subjected to three freeze–thaw cycles, after which the viruses were titered by the Reed-Muench method [24].

### 4.7. RNA Extraction and Quantitative Reverse Transcription-PCR

The EV71 RNA was extracted from infected cells and culture supernatants with TRIzol (Gibco-Invitrogen, Carlsbad, CA, USA) and reverse-transcribed using a PrimeScript RT reagent kit (TaKaRa, Dalian, China) according to the manufacturer’s instructions. The products of reverse transcription were quantified with the SYBR Premix Ex Taq II (perfect real time) kit (TaKaRa) and detected with a Step One Plus sequence detection system (Applied Biosystems, Foster City, CA, USA). Expression of actin was used as an internal standard. The special primer sequences were: EV71-VP1-F: 5'-CACACAGGTGAGCAGTCATCG-3', EV71-VP1-R: 5'-GTCTCAATCATGCTCTC GTCACT-3'; Actin-F: 5'-GGCGGGACCACCATGTACCCT-3', Actin-R: 5'-AGGGGCCGGACTCGT CATACT-3'.

### 4.8. Immunofluorescence Assay

The RD cells infected with EV71 in a 24-well plate were fixed with 4% paraformaldehyde for 20 min and permeabilized with 0.5% Triton X-100 (in PBS) for 20 min, then blocked with 1% bovine serum albumin for 30 min, and subsequently incubated with the primary antibody (mouse anti-enterovirus 71 monoclonal antibody) for 2 h, followed by the appropriate Alexa-Fluor-488-labeled secondary antibody (Alexa-Fluor-488-conjugated AffiniPure goat anti-mouse IgG (H + L)) for 60 min. Cell nuclei were stained with 40, 6-diamidino-2-phenylindole (DAPI). After each step, the slides were washed repeatedly with PBS with Tween (PBST). Fluorescence was observed and recorded using a confocal laser-scanning microscope.

### 4.9. Flow Cytometry Analysis

For the apoptosis assay, RD cells in 6-well plates infected with 100 TCID_50_ of EV71 were left untreated or treated with 40 µg/mL 4s for 36–48 h, until the CPEs of virus control cells reached 70%–80%. The cells were stained with Annexin-V- fluorescein and propidium iodide, according to the manufacturer’s instructions and subsequently subjected to flow cytometry analysis.

## 5. Conclusions

We have found that 18 gramine derivatives exhibited selective inhibitory effects against EV71 infection, while the anti-EV71 activity of the lead compound gramine was not observed. These derivatives mainly acted on the early stages of EV71 replication post infection. Among these derivatives, one compound containing pyridine and benzothiazole units (compound **4s**) demonstrated the highest potency against EV71, and could profoundly inhibit virus-induced CPE, progeny viral yield, viral RNA replication, protein synthesis, and virus-induced apoptosis in RD cells. Our findings indicate that derivative **4s** might be a feasible therapeutic agent against EV71 infection. Moreover, gramine derivatives might provide promising lead scaffolds for the further design and synthesis of potential antiviral agents.
